# Metabolic plasticity of IDH1*-mutant* glioma cell lines is responsible for low sensitivity to glutaminase inhibition

**DOI:** 10.1186/s40170-020-00229-2

**Published:** 2020-10-21

**Authors:** Victor Ruiz-Rodado, Adrian Lita, Tyrone Dowdy, Orieta Celiku, Alejandra Cavazos Saldana, Herui Wang, Chun Zhang Yang, Raj Chari, Aiguo Li, Wei Zhang, Hua Song, Meili Zhang, Susie Ahn, Dionne Davis, Xiang Chen, Zhengping Zhuang, Christel Herold-Mende, Kylie J. Walters, Mark R. Gilbert, Mioara Larion

**Affiliations:** 1 Neuro-Oncology Branch, National Cancer Institute, Center for Cancer Research, National Institutes of Health, 37 Convent Drive, Building 37, Room 1136A, Bethesda, Maryland USA; 2 Genome Modification Core, Laboratory Animal Sciences Program, Frederick National Lab for Cancer Research, National Institutes of Health, Frederick, Maryland USA; 3 Structural Biophysics Laboratory, National Cancer Institute, Center for Cancer Research, National Institutes of Health, Frederick, Maryland USA; 4 Division of Neurosurgical Research, Department of Neurosurgery, University Hospital Heidelberg, Heidelberg, Germany

**Keywords:** IDH1-mutant, Gliomas, Glutaminase, CB839, AGI5198, ^13^C tracing

## Abstract

**Background:**

Targeting glutamine metabolism in cancer has become an increasingly vibrant area of research. Mutant IDH1 (IDH1^*mut*^) gliomas are considered good candidates for targeting this pathway because of the contribution of glutamine to their newly acquired function: synthesis of 2-hydroxyglutarate (2HG).

**Methods:**

We have employed a combination of ^13^C tracers including glutamine and glucose for investigating the metabolism of patient-derived IDH1^*mut*^ glioma cell lines through NMR and LC/MS. Additionally, genetic loss-of-function (in vitro and in vivo) approaches were performed to unravel the adaptability of these cell lines to the inhibition of glutaminase activity.

**Results:**

We report the adaptability of IDH1^*mut*^ cells’ metabolism to the inhibition of glutamine/glutamate pathway. The glutaminase inhibitor CB839 generated a decrease in the production of the downstream metabolites of glutamate, including those involved in the TCA cycle and 2HG. However, this effect on metabolism was not extended to viability; rather, our patient-derived IDH1^*mut*^ cell lines display a metabolic plasticity that allows them to overcome glutaminase inhibition.

**Conclusions:**

Major metabolic adaptations involved pathways that can generate glutamate by using alternative substrates from glutamine, such as alanine or aspartate. Indeed, asparagine synthetase was upregulated both in vivo and in vitro revealing a new potential therapeutic target for a combinatory approach with CB839 against IDH1^*mut*^ gliomas.

## Background

Isocitrate dehydrogenase I gene (IDH1) mutations occur in up to 80% of low-grade (WHO grade II) gliomas and about 5% of all glioblastomas (GBM) [1, 2]. IDH1 is also mutated in acute myeloid leukemia (AML) [3], cholangiocarcinomas, melanomas, chondrosarcomas, fibrosarcoma, and other malignancies [4–6]. Despite being one of the few examples of a metabolic enzyme’s mutation linked directly to cancer and its prevalence, the contribution of IDH1^*mut*^ enzyme activity to global tumor metabolism and its involvement in oncogenesis are not fully understood. The substitution of an arginine to a histidine (R132H) is the most common mutation (90%), and occurs in the active site of the enzyme leading to formation of 2-hydroxyglutarate (2HG) at the expense of α-ketoglutarate (α-KG) and NADPH [7–10]. 2HG has been previously reported to occur naturally [11, 12], although in lower concentrations than those found in IDH-mutated tumors. The carbon source of this metabolite is still under investigation, although reports have linked glutamine (Gln) to 2HG formation [8, 13, 14]. Glucose and Gln are the major carbon sources for tumor cells; although glucose is known to be an essential nutrient for tumor survival and progression, recent investigations have also highlighted the importance of Gln as a major nutrient employed by cancer cells [15–17] participating in different pathways such as (1) synthesis of fatty acids via reductive carboxylation under hypoxia, (2) replenishment of the TCA cycle under conditions of citrate usage as a biosynthetic precursor, providing the majority of oxaloacetate and aspartate, (3) glutathione (GSH) biosynthesis and nitrogen source for nucleotide biosynthesis, a process involved in cell division. In addition, Gln is a N donor for the synthesis of many amino acids [15, 18]. Gln is converted to glutamate (Glu) via the action of glutaminase (GLS) which is encoded by two genes: kidney-type glutaminase (GLS1/KGA), which is also expressed in the brain, and liver-type glutaminase (GLS2) [19]. Inhibiting GLS has been a successful therapeutic strategy in a variety of cancers that are addicted to Gln [19–27]. Since Gln can serve as a carbon source to produce 2HG in IDH1^*mut*^ cancers, GLS inhibitors may provide therapeutic benefit in the treatment of these gliomas. In fact, several reports suggest that the inhibition of GLS preferentially impacts the growth of IDH1^*mut*^ tumors [28, 29]. The effects of GLS inhibitors, such as CB839, on solid tumors harboring IDH1 mutations was studied in at least one clinical trial (NCT0207186230). However, little is known about the contribution of the Gln/Glu pathway to 2HG formation and the metabolic consequences of targeting GLS in IDH1^*mut*^ gliomas. Herein, we have assessed the role of the Gln/Glu pathway on 2HG formation and cell growth in a panel of endogenously IDH1-mutated cell lines. We explored the connection of 2HG synthesis and glutamine and investigated the metabolic consequences of GLS inhibition. We found that our panel of patient-derived IDH1^*mut*^ cell lines experiences a mild effect on proliferation upon treatment with CB839. Therefore, we investigated the metabolic adaptations that enable these cell lines to overcome inhibition of GLS. Upregulation of other Glu-transforming enzymes, among which asparagine synthetase was a common modification to the three cell lines investigated herein, suggests a high ability for metabolic remodeling. Our in vivo models reveal this metabolic compensation of IDH1^*mut*^ glioma against GLS inhibition and provide additional insights on potential combinatorial treatments.

## Methods

### Cell culture

HT1080, TS603, NCH1681 [30], BT142, GSC923, GSC827, and NCH612 [30] were grown in DMEM/F12 medium supplemented with antibiotics, 1% N2 growth supplement, heparin sulfate, EGF and FGF, and FBS (10%) for HT1080 only.

### Dose-response curves and proliferation assays

50,000 to 150,000 cells/well were seeded in 48-well plates with 0.75 mL of culture media in triplicate. CB839 was dissolved in DMSO, and it was added at different concentrations to each well. Cells were then incubated for 72 h; subsequently, neurospheres were mechanically disaggregated and counted using the Vi-CELL XR cell counter. For the proliferation assays, cells were incubated in triplicate for each time point (24, 48, and 72 h) and treatment status, and subsequently counted following the same protocol described above.

### Synergy analysis

Cell number was utilized to assess the inhibitory effect of CB839 and AGI5198 at 16 different combinations for 72 h. Synergy scores were obtained through SynergyFinder 2.0 [31], and the ZIP (zero interaction potency) [32] score was utilized to evaluate the performance of the combination treatment.

### Metabolite extraction

Cells were plated at 5 × 10^6^ per flask (T75) with AGI5198 or CB839 dissolved in deuterated DMSO (d-DMSO) or an equivalent volume of d-DMSO for NMR experiments and regular DMSO for LC-MS. After 72 h, cells were quenched and collected as follows. One millimeter of media was saved for further analysis, and the remaining media were aspirated from adherent cells, washed with PBS, and subsequently by water, and then, 1 mL of ice-cold water was added to the flask and immediately placed onto dry ice. These frozen cell layers were then scraped and transferred to a 15-mL tube and placed into dry ice until metabolite extraction. Neurospheres were collected after these 72 h incubation, centrifuged at 1100 rpm for 5 min, 1 mL of the supernatant was saved, and the remaining media was discarded. This process was repeated twice by adding 5 mL of PBS to wash the cells, and a final washing step carried out using 2 mL of water. Finally, the pellet was re-suspended in 1 mL of ice-cold water and immediately placed into dry ice. For ^13^C tracing experiments, cells were incubated in the same media described above, but without glutamine or glucose, which were added at the same concentration as the original media in the form of ^13^C-U-glutamine/glucose.

### Sample preparation for NMR and LC-MS

Cell suspensions in water were lysed by 3 cycles of freeze-thawing, including a 5 min sonication process in an ice-water bath during the thawing step. Twenty microliters of the homogenate was put aside for protein quantification by the Bradford method. Subsequently, metabolites were extracted by resuspending the cell pellet in a 1:2:2 water:methanol:chloroform solution. Tumor tissue was first weighted as frozen and subsequently homogenized using a bullet blender tissue homogenizer in the same solvent mixture detailed above and further processed in the same way as cell extracts. For LC-MS, methanol was spiked with internal standard (nitrodracylic acid and isocaramidine sulfate). Then, samples were centrifuged at 12,000 rpm, for 20 min at 4 °C. The two resulting phases (upper aqueous polar and lower organic lipid) were separated, and the protein interface was discarded.

For NMR, the top (hydrophilic) layer was then transferred to a vial and dried under a stream of N_2_. The sediment was reconstituted in 550 μL of pH 7 phosphate buffer (100 mM) containing 10% D_2_O (0.05% wt. d-TSP) and 1% NaN_3_, span-down at 10,000 rpm, for 10 min at 4 °C, and the clear supernatant was then transferred to a 5-mm NMR tube. For 1D-^13^C experiments, the protocol was followed as above, but resuspending the dried sediment in a phosphate buffer pH 6, as it was described as the best condition for 2HG observation by ^13^C NMR [33]. For 1d-HSQC experiments, dried sediment was reconstituted in 180 μL of the phosphate buffer described above but in 100% D_2_O and transferred to a 3 mm-NMR tube.

For LC-MS, the two phases (upper aqueous polar and lower organic lipid) were evaporated to dryness under N_2_ gas stream and stored at − 80 °C. All cell extracts were reconstituted in 130 μL of chilled 60:40 MeOH/H_2_O, centrifuged at 13,000 rpm for 10 min at 4 °C, and transferred to a LC-MS vial with fixed glass inserts for analysis. Pooled quality control (QC) samples were prepared by combining 10 μL of each sample.

### NMR spectral acquisition and processing

All the spectra were acquired at 25 °C on a Bruker Avance III 600 MHz spectrometer (Structural Biophysics Laboratory, NCI, Frederick, MD, USA) equipped with a cryogenically cooled probe. Single pulse ^1^H NMR experiments were performed using the *noesygppr1d* (TopSpin 3.5, Bruker Biospin) pulse sequence for water suppression. For each spectrum, 128 scans were acquired, with a relaxation delay of 3 s, a spectral width of 10,800 Hz, and a time domain of 32 K points. Spectra were referenced to the TSP internal standard signal (s, *δ* = 0.00 ppm), zero-filled to 64 K points, phased and baseline-corrected using ACD Labs Spectrus Processor 2016, and an exponential line broadening function of 0.30 Hz was applied. For quantification, ^1^H NMR resonance signals were normalized to the TSP singlet located at 0.00 ppm and corrected to either the total protein content as obtained from the Bradford assay or the cell number.

For ^13^C NMR spectral acquisition, the experimental conditions were 1.5 s delay, flip angle of 45°, and a spectral width of 32 KHz. The number of scans was 18,000. ^1^H decoupling was achieved using WALTZ-16. Free induction decays were zero-filled to 64 K points, phased, baseline-corrected, referenced to the TSP internal standard signal, and multiplied by a weighting function of 1.0 Hz.

1D-HSQC spectra were acquired for 768 scans, a time domain of 3.5 K, a delay of 1.75 s, and a spectral width of 8 KHz. The spectral processing involved the application of exponential line broadening function of 4 Hz and a Gaussian function of 7.5 Hz. All data were processed using ACD Labs Spectrus Processor 2016.

### LC/MS acquisition

Analysis for cell extracts was performed on the Agilent Quadrupole Time-of-Flight Mass Spectrometer coupled with Infinity II 1290 Liquid Chromatography Ultra-High-Pressure unit. LC-MS data acquisition was conducted through three experiments consisting of 3 gradients and two columns to enhance coverage and resolution of amino acid and central carbon metabolites. Global profiling of polar metabolites and relative quantification for steady state, time-dependent ^13^C-label flux of polar metabolites was conducted on both, the AdvanceBio Glycan Map 2.1 × 150 mm 2.7 μm column (Agilent Technologies, California, USA, 683775-913) and Acquity UPLC BEH Amide 2.1 × 100 mm, 1.7 μm column (Waters Corporation, MA, USA, 188004801). Only LC/MS grade solvents and additives were used to prepare mobile phases and wash solutions. Wash cycles consisting of strong wash (50% methanol, 25% isopropanol, and 25% water), weak wash (90% acetonitrile and 10% water), and seal wash (10% isopropanol and 90% water) were implemented to eliminate carryover between injections. Glycan Map column acquisition was performed in both positive and negative electrospray ionization (ESI) modes. Ion separation was accomplished using mobile phase A (10 mM ammonium acetate in 88% water and 12% acetonitrile, pH 6.85) and mobile phase B (10 mM ammonium acetate in 90% acetonitrile, pH 6.85) with column temperature 30 °C at flow rate 0.3 mL/min while executing gradient conditions: 100% B, 0.5 min; 95% B, 2.0 min; 60% B, 3.0 min; 35% B, 5 min; hold 0.25 min; 0% B, 6 min; hold 0.5 min; 100% B, 7.25 min. The mass analyzer acquisition conditions were as follows: drying gas temperature, 325 °C; sheath gas temperature, 250 °C; nebulizer pressure, 45 psig; skimmer voltage, 50 V; octopole radio frequency, 750 V. In ESI positive mode experiment, ms spectra were acquired using a voltage gradient of capillary 3500 V, nozzle 2000 V, and fragmentor 165 V. In ESI negative mode experiment, mass spectra were acquired using a voltage gradient of capillary 3000 V, nozzle 2000 V, and fragmentor 125 V. BEH amide column acquisition was performed under low pH conditions in negative ESI mode only. Feature separation was accomplished using mobile phase A (100% water and 0.1% formic acid) and mobile phase B (100% acetonitrile and 0.1% formic acid) with column temperature 40 °C at flow rate 0.4 mL/min while executing the following gradient conditions: 98% B, 0.75 min; 80% B, 3 min; 30% B, 8 min; 25% B, 9 min; 98% B, 9.5 min. The mass analyzer acquisition conditions were sheath gas temperature 325 °C, drying gas temperature 225 °C, nebulizer 45 psig, skimmer 50 V, octopole radio frequency 750 V, capillary 3000 V, nozzle 2000 V, and fragmentor 125 V.

### LC/MS data analysis

During acquisition, the m/z for each precursor ion was detected at an acquisition rate of 6 spectra/s and 166.7 ms/spectrum over a mass range of 70 to 1100 m/z. Prior to preprocessing datasets, pooled QC samples (TIC, BPI, and EIC) were chromatographically examined to inspect consistency of retention time and signal intensity. QC data showed no evidence of signal degradation. Following acquisition, m/z spectra binning was performed by partitioning the m/z vs. retention time (RT) matrices into fixed width using Agilent Masshunter Profinder B.08.00. Bins were manually inspected to confirm consistent, reproducible integration for all compounds of interests across all samples. Precursor m/z for each bin was determined using molecular feature extraction algorithm to deconvolute, integrate, and envelope parent ions, adducts, natural isotopes, and neutral losses in order to define a composite spectrum. Parameters for the input data range and alignment were restricted to 0.150 to 7.5 min, ion species (H^+^, Na^+^, K^+^, –H_2_O, –H, 2 M-H, 2 M+H), and charge state +1. Ion selection and alignment was configured at 2.0 mDa mass range, and ± 0.2 min retention time. Resulting mass features were annotated based on neutral mass using available databases (Metlin/HMDB) and in-house library of commercial standards. The Vista Flux software was used for the analysis of ^13^C isotopologue incorporation and natural abundance normalization.

### Statistical analysis

Metabolomics data was analyzed using the R statistical software and MetaboAnalyst 3.0 [34].

### RNA extraction and sequencing

Total RNA was extracted from cell lines using PureLink RNA Mini kit (Thermo Fisher Scientific) following manufacturer instructions. RNA quality was measured on an Agilent 2100 Bioanalyzer using the RNA 6000 Pico kit (Agilent Technologies). The transcriptome library was prepared using Illumina TruSeq Stranded mRNA Library Prep kit following the manufacturer’s instructions. The library was sequenced on Illumina HiSeq 3000 using 150 bp paired-end reads targeting an average coverage of 25 million reads per sample.

### RNA-seq data analysis

Strand-specific RNA-seq reads were analyzed using CCBR Pipeliner, which comprises sequencing reads QC, genomic mapping, differentially expressed gene identification. Specifically, CCBR Pipeliner is composed of several tasks: pre-alignment QC, read grooming, alignment, post-align QC, quantification, and differentially expressed gene identification. Initially, sequencing reads that passed QC thresholds were trimmed of adaptor sequences and aligned to the human genome (hg38), and STAR was then used for genomic alignment. The transcripts were quantified using salmon and rsem, and differentially expressed genes were determined using edgeR, DESeq2, and limma/voom. In this analysis we derived differentially expressed genes using thresholds of FDR ≤ 0.05 and fold changes ≥ 1.5 or ≤ − 1.5 across all three methods (for some contrasts *p* ≤ 0.05 and fold change 1.3 or ≤ − 1.3). The functional annotation was carried out using the Ingenuity Pathway Analysis (IPA, www.qiagenbioinformatics.com) software.

### ASNS knockdown

ASNS and GLS Dicer-Substrate Short Interfering RNAs (DsiRNAs) and Scrambled Negative Control DsiRNA were purchased from Integrated DNA Technologies. One million cells were electroporated with DsiRNAs (300 nM for each) or control DsiRNA with Lonza P3 Primary Cell 4D-Nucleofector^TM^ X Kit (V4XP-3024, 100 μL format, program: CA137). All experiments involving these cells were performed in less than a week from electroporation.

### ASNS knockout

To establish ANSN knock out cell line, ASNS guide RNA sequence was cloned into pLenti-CRISPR v2 plasmid. LentiCRISPR v2 was a gift from Feng Zhang (Addgene #52961) [35]. sgRNA sites targeting the ASNS gene were designed using the sgRNA Scorer 2.0 algorithm [36], with the following spacer sequences used for knockout experiments: ATTGTCATAGAGGGCGTGCA (coding exon 3) and ATCGAACTACTGCTGCCCA (coding exon 8). The gRNA plasmids were packaged into lentivirus particles using lipofectamine 2000 (Thermo Fisher) in 293FT cells (Thermo Fisher) with psPAX2 (Addgene #12260) and pMD2.G (Addgene #12259) as previously described [37]. The lentivirus was concentrated with Lenti-X concentrator (Takara) and applied to NCH1681 cell line. Cells were selected under 1 μg/mL puromycin. The genetic knockout of ASNS was tested by western blotting.

### Protein expression analysis

ASNS, GLS, and GPT2 levels were evaluated via western blots. Protein extraction was performed using proteinase inhibitor-containing RIPA lysis buffer. About 15 μg extracted protein was used for western blotting with anti-ASNS antibody (Abcam, ab126254, 1:1000), anti-GPT2 (Abcam, ab232963, 1:1000), anti-GLS (Proteintech, 12855-1-AP, 1:5000), and anti-β-actin antibody (Sigma, A5441, 1:1000) as loading control. Western blotting results were detected by enhanced chemiluminescence substrate (R1004, Kindle Biosciences) and imaged by KwikQuant Imager (D1001, Kindle Biosciences). Band intensities were analyzed by ImageJ.

### Animal studies

All animal experiments were performed in female, 6- to 8-week-old immunodeficient NSG mice (NOD.Cg-Prkdc<scid> Il2rg<tm1Wjl>/SzJ, The Jackson Laboratory, ME), in accordance with the principles and procedures outlined in the NIH Guide for the Care and Use of Animals and approved by the Animal Care and Use Committee of the National Institute of Health (Protocol NOB-008). Cells were subcutaneously injected with (5 × 10^6^ cells in 100 μl HBSS for each mouse) in the right flank. Xenograft size was measured with a sliding caliper twice a week, according to the formula: TV = (width)^2^ × (length)/2. When tumors reached a minimum of 100 mm^3^, animals were randomized into two groups: vehicle controls and CB839 treatment. Following the treatment with or without CB839 (160 mg/kg, oral gavage, twice a day), the mice were euthanized, and the tumor xenografts were harvested 4 h after the last treatment. The tumor tissues were then divided for two parts. One was reserved in − 80 °C immediately for metabolomics, and the other was fixed with 4% paraformaldehyde for histological and immunochemistry analysis.

## Results

### Glutamine is a precursor of 2HG in vitro

**Fig. 1:**
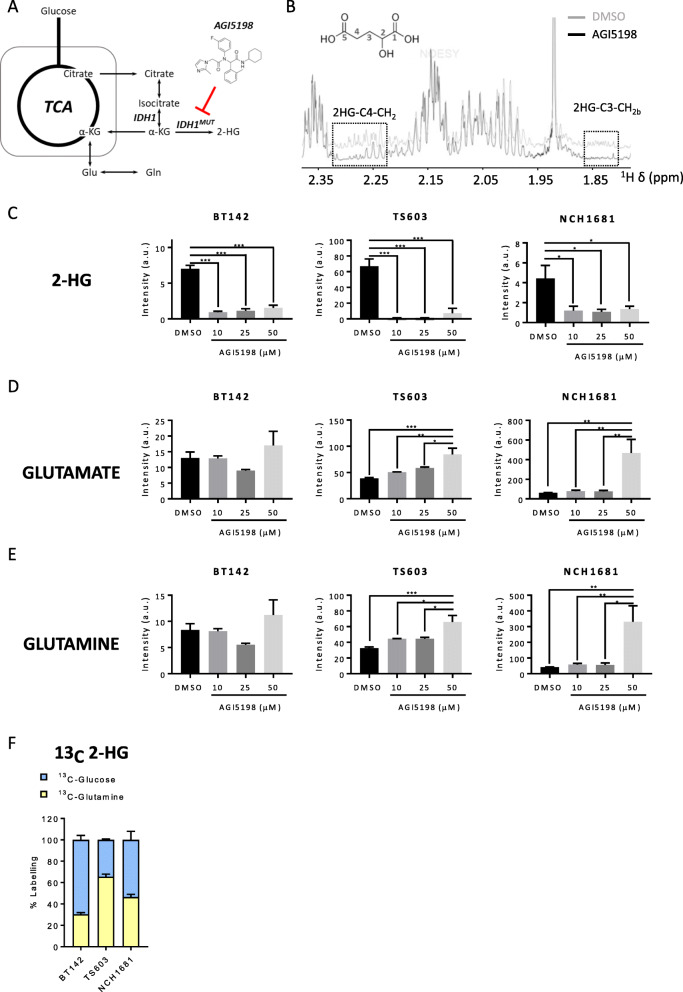
**a** Simplified representation of the TCA cycle and its link with IDH1 enzyme and glutamine. **b** ^1^H NMR representative spectrum of an IDH1^*mut*^ glioma cell line after 10 μM AGI5198 treatment for 72 h (black) or DMSO (gray). The 2HG chemical structure is displayed (upper left insert) with carbon atom numbering included. Boxed areas denote regions for the two protons linked to C4 and one to the C3 (Hb) group, since the remaining one (Ha) is overlaid by the larger proline resonance centered at 1.98 ppm. **c** 2HG, **d** glutamine, and **e** glutamate intensities normalized to protein content from polar extracts of IDH1^*mut*^ cell lines after 10, 25, and 50 μM AGI5198 treatment for 72 h or DMSO obtained by LCMS analysis. Bar plots depicting the normalized intensities from *n* = 3 samples per experiment. *p < 0.05; **p < 0.005; ***p < 0.001 from a one-way ANOVA followed by Tukey’s HSD test for multiple comparisons. **f** Contribution of glucose and glutamine to 2HG formation obtained by LCMS analysis from 2 independent experiments involving the aforementioned ^13^C probes. Bar plots displaying mean ± SD from adjusted percentages (*n* = 3)

First, we evaluated the correlation between the global metabolism of patient-derived IDH1^*mut*^ cell lines and 2HG production by treating our cell lines with AGI5198 (Fig. 1a), a specific inhibitor of the IDH1^*mut*^ protein. Our panel of IDH1^*mut*^ cell lines involved TS603 (anaplastic oligodendroglioma with a 1p19q co-deletion) [38], BT142 (grade III oligoastrocytoma), NCH1681 (grade III astrocytoma) [30], and HT1080 (fibrosarcoma). We detected some metabolic changes attributable to this treatment, including the expected reduction in 2HG (Fig. 1 b and c and Additional Figure 1A) that is readily noticeable by ^1^H NMR as the lowered intensity of the resonance signal centered at 1.83 ppm, since the resonance centered at 2.26 ppm overlaps with that attributable to α-aminoadipate (C5-H_2_) [39], and an increase in the Glu and Gln levels for some cell lines (Fig. 1 d and e). Both Glu and Gln levels were increased as cells were treated with higher concentrations of AGI5198, mainly for TS603 (Fig. 1 d and e) and HT1080 (Additional Figure 1A). 2HG levels do not experience any significant decrease upon AGI5198 concentrations above 10 μM (Fig. 1c). A negligible effect was observed in growth and viability (Additional Figure 1C) of glioma cell lines upon treatment with 10 μM AGI5198 for 72 h in agreement with previous investigations involving models of chondrosarcoma [40] and fibrosarcoma [41], even a positive effect on proliferation has been recently reported in BT142 [42]. This observation may reveal that the inhibition of 2HG synthesis does not correlate to the regulation of cell growth and proliferation.

NCH1681 experiences an increase in Glu/Gln levels only at the highest AGI5198 concentration (Fig. 1 d and e), and they did not experience any significant change for BT142, a cell line which synthetizes the majority of 2HG from glucose, similarly to NCH1681 (Fig. 1f). Contrarily, those cell lines mainly utilizing Gln as 2HG substrate, i.e., TS603 and HT1080 (Fig. 1f and Additional Figure 1B), revealed an upregulation of these amino acids that is correlated with AGI5198 concentration. We further verified the contribution of Gln to 2HG formation and its correlation with AGI5198 treatment via ^13^C-NMR (Additional Figure 1D) by incubating our cells in media containing 2.5 mM ^13^C-U-Gln for 72 h and following the protocol described by Pichumani et al. [43]. The absence of ^13^C resonance signals after AGI5198 treatment confirms that Gln can serve as a 2HG precursor, and by inhibiting IDH1^*mut*^, we can observe an increased availability of both Glu and Gln.

### CB839 inhibits GLS causing a moderate antiproliferative effect on IDH1^*mut*^ cell lines

**Fig. 2:**
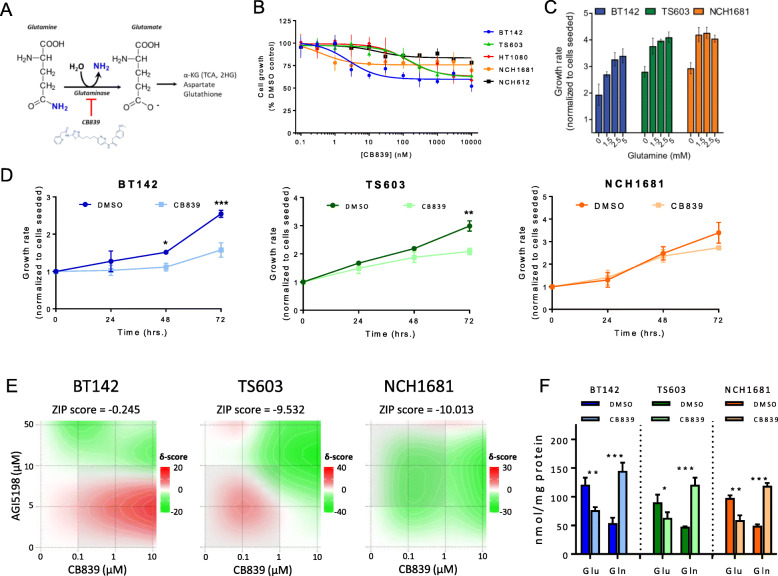
**a** Reaction involving GLS and the inhibition via CB839. **b** Dose response curve of IDH1^*mut*^ glioma cell lines and IDH1^*mut*^ fibrosarcoma (HT1080) against increasing concentrations of CB839. **c** Growth rate of patient-derived IDH1^*mut*^ glioma cell lines at different concentrations of Gln in cell culture media. **d** Growth rate of patient-derived IDH1^*mut*^ glioma cell lines over time upon addition of 1 μM CB839 (data displayed as mean ± SD, *n* = 3 for each time point). **e** Synergy analysis for the 3 patient-derived IDH1^*mut*^ glioma cell lines under 16 different combinations of CB839 and AGI5198 for 72 h based on growth inhibition (*n* = 3 for each combination). Data displayed as a heat map for the visualization of delta scores for the dose regions. ZIP synergy scores included at the top of each heat map for each cell line. **f** Quantification of Glu and Gln *via* ^1^H NMR under CB839 treatment vs DMSO controls. (*n* = 3 for each concentration, drug or time point tested, data displayed as mean ± SD; *p < 0.05; **p < 0.005; ***p < 0.001 from a *t* test unless otherwise indicated)

CB839 is a potent and selective inhibitor of splice variants of GLS1, an enzyme involved in the transformation of Gln into Glu (Fig. 2a). We found that Gln is a main source of 2HG in some of the cell lines investigated herein, and therefore, our cell lines may require an additional supply of Gln-derived Glu to meet their metabolic requirements. Additionally, previous investigations tested CB839 in a clinical trial (NCT02071862 30) for IDH1^*mut*^ glioma. We therefore treated IDH1^*mut*^ cell lines with different concentrations of CB839 for 72 h to assess the effect on both cell growth and viability of this agent. We observed an antiproliferative effect (Fig. 2b) which was cell line dependent, being more intense for BT142 (an average 40% growth reduction at 1 μM CB839) and showing only a 15% growth reduction for NCH612. We then explored whether our IDH1^*mut*^ glioma patient-derived cell lines, i.e., TS603, BT142, and NCH1681 experienced the same antiproliferative effect under Gln-deprivation conditions (Fig. 2c) for 72 h. BT142, TS603, and NCH1681 growth rate was reduced in media without Gln, although recovered at 1.5 mM; however, BT142, the most sensitive cell line, required 2.5 mM (standard concentration in DMEM/F12 media) to reach its maximal growth. Concentrations above standard conditions (2.5 mM) did not increase the growth rate of the cell lines. BT142 also displayed an earlier significant antiproliferative response (24–48 h) compared to TS603 and NCH1681 (Fig. 2d). However, viability was not affected from the treatment (Additional Figure 2A). Furthermore, we explored the combination therapy of CB839 and AGI5198 (Fig. 2e) through a synergy analysis based on ZIP reference model [32]. ZIP scores revealed an antagonistic effect for TS603 and NCH1681, since their scores were close or below − 10 [31]; contrarily, the effect on BT142 was a mild additive effect. These results indicate an increased proliferation in the combination treatment compared to CB839 alone, in accordance with a decreased need of Gln for those IDH1^*mut*^ cells mainly utilizing Gln for 2HG synthesis (Fig. 1f), i.e., NCH1681 and TS603, which concomitantly experienced an inhibition on 2HG synthesis by AGI5198. We then quantified intracellular Glu and Gln upon 1 μM CB839 treatment to confirm the expected inhibitory effect on GLS. We observed a significant decrease in Glu levels and increased Gln ones (Fig. 2f). These changes were accompanied by the downregulation of Glu downstream metabolites such as aspartate (Asp), n-acetyl-aspartate (NAA), and GSH (Additional Figure 2B) revealing the characteristic metabolic profile of GLS inhibition [27]. Notably, this reduction in GSH upon CB839 [44] is involved in the foundation for a clinical trial testing CB839 when combined with radiotherapy/temozolomide in IDH mutant grade II/III astrocytoma (NCT03528642).

### The TCA cycle is dysregulated in IDH1^*mut*^ cell lines upon CB839 treatment

**Fig. 3:**
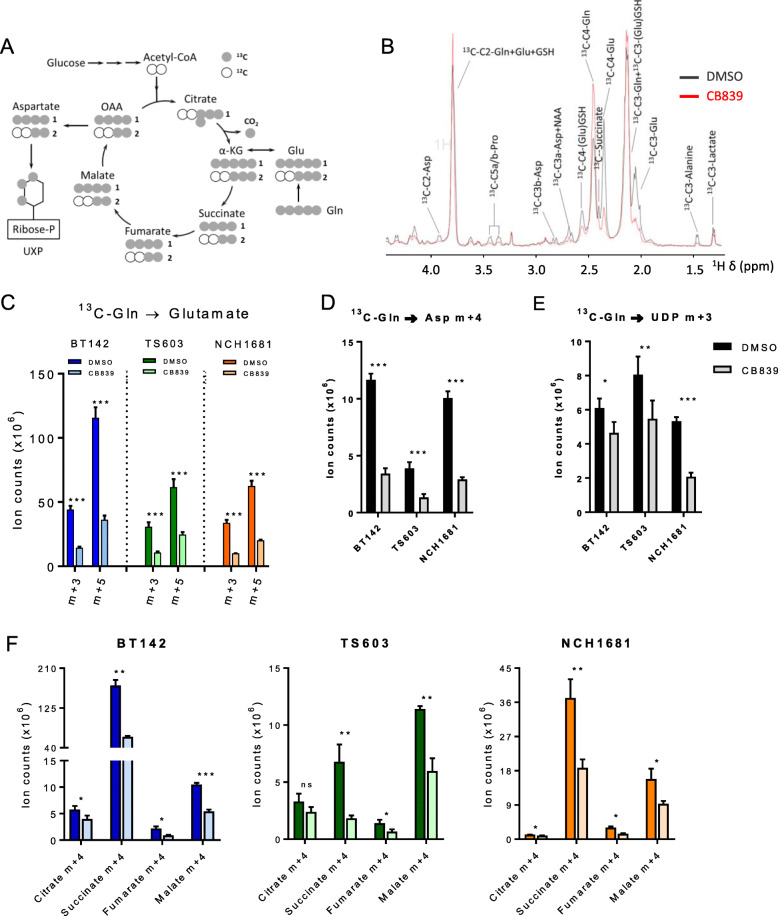
**a** ^13^C tracing map from U-^13^C-Gln into the TCA cycle and derived metabolites (gray circles = ^13^C, white circle = ^12^C). **b** 1D HSQC representative spectrum of an IDH1^*mut*^ glioma cell line under DMSO (black) or CB839 (red) treatment displaying those resonances arising from ^13^C labeling from 72 h incubation in cell media containing ^13^C-U-Glutamine. **c** Intensities from isotopologues m+3 and m+5 of Glu under CB839 treatment (72 h) arising from ^13^C-Gln incorporation. ***p < 0.001. **d** ^13^C-Gln contribution to Asp (m+4), UDP (m+3) (**e**) and representative TCA cycle intermediates (m+4 isotopologues) (**f**). (*n* = 3, data displayed as mean ± SD; *p < 0.05; **p < 0.005; ***p < 0.001 from a *t* test). m+0, unlabeled; m+1, m+2, m+3, m+4, m+5, and m+6 represent the degree of m/z increase due to ^13^C labeling

The TCA cycle serves the cell as a major provider of intermediates for nucleotide synthesis [45] and generates energy for cellular processes [46]. One of the main entries into this cycle is the route initiated with the transformation of Gln into Glu and the subsequent oxidative deamination to α-KG and ammonia. Since the first reaction is catalyzed by GLS, we examined the metabolic consequences of its inhibition by CB839 through ^13^C-U-Gln tracing experiments via LC-MS and NMR. Initially, we checked ^13^C-Gln incorporation (Fig. 3a) into downstream metabolites upon CB839 treatment, revealing the expected decreased levels from a treatment with a GLS inhibitor (Fig. 3b). As expected, reduced Gln flux towards Glu was observed upon CB839 treatment (Fig. 3c), specifically in the 2 most abundant isotopologues, m+3 and +5. Incorporation of ^13^C-Gln into Asp (m+4) and UDP (m+3) was also reduced due to GLS inhibition (Fig. 3 d and e) in addition to Gln-derived metabolites involved in the TCA cycle such as citrate, succinate, fumarate, and malate (Fig. 3f). We observed significant reduction in the m+4 isotopologue derived from ^13^C-Gln in the first round of the TCA cycle, which was indeed the most abundant isotopologue for all the TCA cycle metabolites detected, revealing a global reduction of Gln flux into the cellular metabolism for all reactions that require a previous conversion of Gln into Glu. We also explored the contribution of Gln to wild-type glioma cell lines through the same ^13^C-tracing approach. Both GSC827 and 923 cell lines displayed a lower incorporation of Gln into the TCA cycle metabolites than the IDH1^*mut*^ cell lines utilized in these experiments (Additional Figure 3).

### Potential compensatory mechanisms of GLS inhibition

**Fig. 4:**
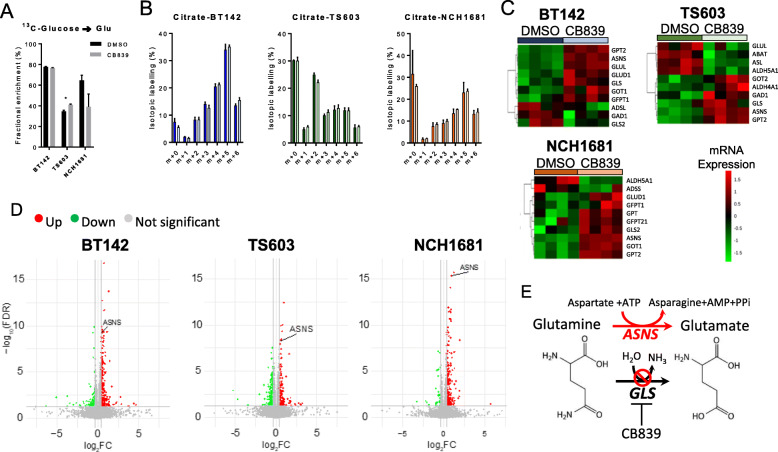
**a** ^13^C incorporation into Glu from ^13^C-U-glucose (*n* = 3, data displayed as mean ± SD; *p < 0.05 from a *t* test). **b** Distribution of isotopologues of citrate when cells were grown in media containing ^13^C-U-glucose (m+0, unlabeled; m+1, m+2, m+3, m+4, m+5, and m+6 represent the degree of m/z increase due to ^13^C labeling). **c** Heatmaps displaying the top 10 differentially expressed mRNAs included in the glutamate pathway. **d** Volcano plots depicting the -Log(FDR *p* value) vs log2 fold-change (FC) for the mRNA transcripts analyzed. Gray dots refer to those mRNA which have not reached statistical significance (FDR *p* value < 0.05) nor delivered a Log2FC> 1 or <− 1. Those colored in green are downregulated after treatment, and the red ones are upregulated. **e** Diagram displaying the correlation between ASNS activity and CB839 metabolic effect

In view of the moderate effect on cell growth of CB839 on our patient-derived IDH1^*mut*^ glioma cell lines, we explored the potential metabolic adaptations that these cells may acquire in order to overcome the reduced flux from Gln to Glu. We first assessed the glucose metabolism of these cells through ^13^C-U-glucose tracing experiments since recent reports have described a dynamic glycolytic activity in IDH1^*mut*^ tumor cells [47, 48]. We observed the absence of any major upregulation of glucose flux through Glu in BT142 (Fig. 4a), but there was an increase in the fractional labeling of Glu from ^13^C-U-glucose for TS603 (*p* = 0.02) and a decrease for NCH1681 (*p* > 0.05). The difference between untreated and CB839-treated cells was negligible with regard to ^13^C-U-glucose incorporation into the TCA cycle as there was not an enhanced incorporation of ^13^C into the citrate pool (Fig. 4b) nor further TCA cycle metabolites (Additional Figure 4A). This ^13^C tracing experiment also revealed that BT142 and NCH1681 display a high activity of pyruvate carboxylase entry into the TCA cycle, since the main citrate isotopologue was m+5. Increased activity of pyruvate carboxylase has been reported as a potential metabolic mechanism for tumor growth in situations where GLS activity is challenged [49, 50], although growing the cells in glucose-free media (Additional Figure 4B) for 72 h did not affect significantly the viability nor the growth of CB839-treated cells. In order to gain more insight into the overall metabolic changes arising from GLS inhibition, we performed global RNAseq analysis upon 1 μM CB839 treatment. We observed an upregulation of different enzymes involved in Glu metabolism (Fig. 4c), i.e., GPT2, the enzyme involved in the transamination between alanine and pyruvate, yielding Glu. Nevertheless, the fold change (FC) computed from the RNAseq analysis did not yield values above the threshold displayed in the volcano plot, although statistical significance was attained (after FDR correction) for the 3 cell lines. Moreover, ^13^C-glucose-derived alanine m+3 levels were decreased upon CB839 treatment in TS603 (Additional Figure 4C), which may indicate a conversion of this amino acid into α-KG in order to increase the Glu pool. GOT (glutamate oxaloacetate transaminase), a transaminase which involves the interconversion of Asp+α-KG with oxaloacetate+Glu, located either inside the mitochondria (GOT2) or the cytosol (GOT1) were upregulated in the 3 cell lines; GOT2 in TS603 and the cytosolic isoform (GOT1) in BT142 and NCH1681. We also observed a common trend in our 3 cell lines involving an upregulation of asparagine synthetase (ASNS) (Fig. 4d), the enzyme which uses Asp and Gln to yield asparagine (Asn) and Glu consuming ATP. We also analyzed the levels of both ASNS and GPT2 after CB839 treatment, which revealed a slight upregulation of these enzymes, at the protein expression level in vitro (Additional Figure 4D), although not attaining statistical significance. The upregulation of ASNS when Gln utilization is compromised is in accordance with previous investigations [51, 52]. Hence, this adaptation may serve as a bypass of the GLS reaction in order to generate Glu utilizing aspartate as substrate (Fig. 4e).

### ASNS contributes to metabolic adaptation to CB839 treatment in vitro and in vivo

**Fig. 5:**
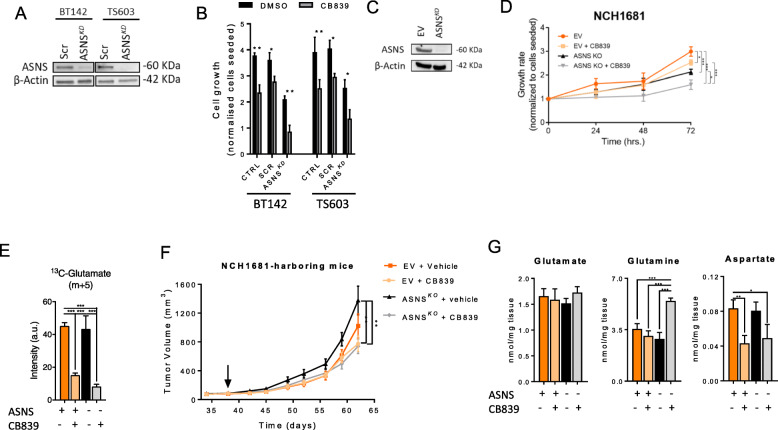
**a** Western blot of ASNS after knocking down the gene. **b** Growth of BT142 and TS603 for parental cells (CTRL), SCR (siRNA control), and ASNS^*KD*^ under CB839 or DMSO treatment for 72 h. **c** Western blot of ASNS in NCH1681-ASNS^*KO*^ cells. **d** Growth of NCH1681 and the derived ASNS^*KO*^ upon CB839 treatment throughout 3 days (for purposes of clarity, only the proliferation differences at 72 h were statistically assessed, *n* = 3 samples per experiment and time point. *p < 0.05; **p < 0.005; ***p < 0.001 from a one-way ANOVA followed by Tukey’s HSD test for multiple comparisons). **e** ^13^C tracing of NCH1681-ASNS^*KO*^ or empty vector cells upon CB839 treatment incubated in media containing ^13^C-U-glutamine for 72 h (*n* = 3, data displayed as mean ± SD; *p < 0.05; **p < 0.005; ***p < 0.001 from a one-way ANOVA followed by Tukey’s HSD test for multiple comparisons). **f** Tumor volume of the mouse models generated after injection in the flank of the cell lines displayed in **c**. The arrow points at the starting treatment time. (*n* = 8–12, for each time point and class), data displayed as mean ± SEM (*p < 0.05; **p < 0.005 from a two-way ANOVA by Tukey’s HSD test for multiple comparisons). **g** Glutamate, glutamine, and aspartate levels for the tumor tissue collected from the mouse models after 27 days of CB839-treatment or vehicle (*n* = 3, data displayed as mean ± SD; *p < 0.05; **p < 0.005; ***p < 0.001 from a one-way ANOVA followed by Tukey’s HSD test for multiple comparisons)

We generated ASNS knockdowns in BT142 and TS603 cell lines (Fig. 5a) in order to investigate the effects of blocking the common metabolic adaptation of IDH1^*mut*^ cells to GLS inhibition. The cell growth of both knockdowns was affected even in the absence of CB839 when compared to the parental cell line (Fig. 5b). BT142 and TS603 lacking ASNS showed inhibition of cell proliferation, suggesting a major role for this enzyme in bypassing the GLS reaction and lowering the sensitivity to CB839. We also generated a stable knockout on the most CB839-resistant cell line, i.e., NCH1681 (Fig. 5c), and firstly evaluated the proliferation of this ASNS^*KO*^ in vitro. Knocking out ASNS sensitized this cell line to CB839 treatment (Fig. 5d); indeed, conversion of Gln into Glu was decreased in both the ASNS^*KO*^ and the empty vector upon CB839 treatment, and the lowest levels of Glu m+5 were observed in the ASNS^*KO*^+CB839 samples (Fig. 5e). In order to investigate the involvement of ASNS in a potential resistance mechanism against GLS inhibition in vivo, we generated a mouse xenograft of NCH1681. Preliminary experiments were set to determine if ASNS overexpression was maintained in vivo upon CB839 treatment. We explored this treatment in our mouse model for 1 week, and we did not observe any reduction in tumor volume neither in Glu and Gln levels (Additional Figures 5A and B); however, this short treatment revealed the upregulation of ASNS in tumor tissue (Additional Figures 5C and D). Therefore, we generated a mouse model of NCH1681-ASNS^*KO*^ and evaluated the effect of CB839 in a longer treatment (Fig. 5f). Tumor volume of the NCH1681-ASNS^*KO*^ displayed the highest volume after 65 days, which was significantly different to both treated models (empty vector, EV, and the KO). Extending the treatment did not cause a reduction of tumor size or weight (Additional Figure 6A) in the EV CB839-treated animals. These mouse models generated from injections of IDH1^*mut*^ glioma cell lines into the flank were subjected to metabolomics analysis after 4 weeks of treatment with either CB839 or vehicle. We did not observe any change in Glu concentration, but Gln levels were significantly higher in the ASNS^*KO*^+CB839 group (Fig. 5g). While CB839 decreased Asp levels in tumor tissue of both mice cohorts (which either expressed ASNS or not), knocking out ASNS did not affect Asp levels upon CB839 treatment. As an additional control, we analyzed the tumor levels of Asn in vivo, which provided further evidence of ASNS-knocked out, since Asn concentration was significantly reduced in the KO when compared to the EV group within the vehicle-treated condition (Additional Figure 6B).

## Discussion

IDH1-mutant tumors display a characteristic metabolic phenotype which has been suggested to better respond to GLS inhibition [28] than its wild-type counterpart. Recent investigations have reported how overexpression of IDH1^*mut*^ protein in glioma cell lines downregulates TCA cycle intermediates [53] in view of the Gln demand for synthetizing 2HG and have explored the inhibition of GLS as a therapeutic approach against IDH1^*mut*^ cancers [54, 55]. Therefore, we tested whether CB839, a newly-developed GLS inhibitor, had any effect on patient-derived IDH1^*mut*^ cell lines in vitro and in vivo. Gln is a metabolite mainly involved in the synthesis of Glu, which can be further metabolized into other amino acids, enter the TCA cycle, or generate GSH; in fact, Glu is known to be involved in a large number of pathways [56]. We have conducted an untargeted metabolic profiling of patient-derived IDH1^*mut*^ cell lines upon AGI5198 treatment in order to unravel the potential connectivity of 2HG with the basal metabolism, finding that Glu and Gln were affected by IDH1^*mut*^ inhibition in a cell line-dependent fashion. This correlation was more evident in the cell lines which use Gln as the main precursor of 2HG. In our in vitro experiment, ^13^C-Gln incorporation into 2HG ranges from 30 to 70% of the total metabolite pool in our cell lines, contrarily to the results published by Garrett et al. showing that the majority of 2HG arises from Gln [53] and those reported in vivo in a genetically engineered mouse model of IDH1^*mut*^ glioma [48], although earlier in vitro investigations have also reported a poor incorporation of Gln into the 2HG pool [28, 29]. Interestingly, our data complement previous reports revealing a decrease in Glu and Gln after transfection of glioma cell lines with the IDH1^*mut*^ gene [57]. We have observed how combining AGI5198 and CB839 generated an increased proliferation compared to the GLS inhibition alone. That observation confirms that the increased availability of Gln attributable to the inhibition of the IDH1^*mut*^ enzymatic activity partially protects the cells against potential antiproliferative effects of a GLS inhibitor. Inhibition of 2HG synthesis might cause a redirection of Gln carbon towards the TCA cycle and thus remove the stress imposed on the C pools by the IDH1 mutation. Our data suggest that CB839 effect on patient-derived IDH1^*mut*^ cell lines is not as intense as previously reported in other IDH1-mutant tumors due to the metabolic plasticity of these cells. Specifically, our glioma cell lines overexpress ASNS among other Glu-utilizing enzymes which have the ability to generate Glu, bypassing the GLS reaction (Fig. 4e). This adaptation allows the cells to sustain proliferation both in vivo and in vitro. Metabolic fate of asparagine in mammals is exclusively focused on protein synthesis [58] since most cellular types do not express asparaginase [59]; therefore, they cannot synthetize Asp from Asn. The knockdown of ASNS along with GLS inhibition completely halted the growth of these cells in vitro highlighting the upregulation of ASNS as a major contributor to the metabolic response against CB839 treatment. We believe that the conversion of aspartate (derived from alternative sources to Gln and glucose) into Asn-yielding Glu contributes to the compensatory response to GLS inhibition aiming at increasing the Glu cellular pools yet diminished (Fig. 2g). Interestingly, rescue experiments treating our cells with Asn did not reverse the antiproliferative effect of CB839 (Additional Figure 4F). Intriguingly, the in vivo experiments showed an enhanced effect of CB839 within the ASNS^KO^ animal models but without any significant difference when both CB839-treated groups are compared. Therefore, additional compensatory mechanisms may exist, since Glu levels were unaffected. We did observe a combination of upregulated metabolic enzymes (Fig. 4d) related to Glu pathways which can all contribute to maintain the Glu pool stable, even in GLS-inhibition conditions along with absence of ASNS. Hence, it is of major importance to consider the rather complicated mingling of metabolic pathways when targeting a specific route. Further metabolic compensations upon GLS inhibition have been also previously reported such as lipid catabolism and autophagy [60]. Additionally, a recent investigation described a whole set of different pathways and additional enzymatic reactions involved in the compensatory response of pancreatic cancer against CB839 [61] including branched chain aminotransferase 1 (BCAT1), gamma-glutamyl hydrolase (GGH), and 5-oxoprolinase (OPLAH). We opted for this xenograft model to overcome the potential drug delivery problems that might have arisen from poor BBB penetration of CB839 [44]; furthermore, mouse xenografts of the breast [27], pancreas [61], osteosarcoma [62], and glioma [44] have demonstrated an efficient delivery of CB839 into those tumoral tissues.

## Conclusions and limitations of the study

Here, we show that, despite the previously reported sensitivity of IDH1^*mut*^ gliomas to GLS inhibition, our patient-derived IDH1^*mut*^ cell lines can overcome that inhibition by adapting their metabolism. Rescuing experiments involving adding Asp to the media to yield Asn thus generating Glu are difficult to conduct, since they present limitations due to poor membrane permeability [63] and the potential of direct conversion of Asp into Glu through aspartate transaminase.

The re-wiring of metabolic pathways upon treatments which compromise major routes of metabolism plays a critical role in the search for therapeutic strategies against glioma. Therefore, a combinational approach targeting both GLS and ASNS may deliver improved results on IDH1^*mut*^ gliomas that might not respond solely to GLS inhibition.

## Supplementary information

**Additional file 1: Figure 1.** (A) 2HG, Glutamate and Glutamine levels of HT1080 obtained from the integration of the NMR resonance signals at the 1.80-1.88, 2.33-2.38 and 2.44-2.48 spectral buckets respectively. Intensities were sum-normalized for each spectra. AGI5198 treatment was conducted for 72 hrs. n=3 samples per experiment. *, *p*<0.05; **, *p*<0.005; ***, *p*<0.001 from a one-way ANOVA followed by Tukey's HSD test for multiple comparisons. (B) Different contribution of glucose and glutamine to 2HG in HT1080. ^13^C tracing experiment performed by LCMS including ^13^C-U-Glucose or ^13^C-U-Glutamine for 72 hrs. (C) Viability of IDH1^*mut*^ glioma cell lines upon 10 μM AGI5198 for 72 hrs. (D) ^13^C spectral region for 2HG identification including assignments. C4D45, doublet resonance signals at 33.6 ppm and 34.1 ppm from coupling between C4 and C5; C4S, singlet at 33.8 ppm, arising from C4.

**Additional file 2: Figure 2.** (A) Viability of IDH1^*mut*^ glioma cell lines upon 1 μM CB839 for 72 hrs. (B) Levels of glutamate downstream metabolites after treatment with 1 μM CB839 for 72 hrs.

**Additional file 3: Figure 3.** Fractional enrichment of TCA cycle metabolites in glioma cell lines both IDH mutant and wild type via LCMS. Cells were grown in media containing ^13^C-U-Glutamine for 72 hrs. n=3, bar plots depicting mean ± SD. Significance markers (ns, not significant; **, *p*<0.005; ***, *p*<0.001) arising from a t-test of the averaged percentages of IDH1^*mut*^ vs IDH1^*wt*^.

**Additional file 4: Figure 4.** (A) ^13^C labelling of TCA cycle metabolites from ^13^C-U-glucose as isotopologue % over the total pool of the metabolite (n=3, bar plots depicting mean ± SD). (B) Growth of IDH1^*mut*^ glioma cell lines upon treatment with CB839 and in glucose (gluc) deprivation conditions (n=3, bar plots depicting mean ± SD). (C) Levels of alanine m+3 from ^13^C-U-glucose in TS603 cell line upon treatment with 1 μM CB839 for 72 hrs (n=3, bar plots depicting mean ± SD, ***, *p*<0.001, from a t-test). (D) Western Blot of ASNS and GPT2 for the 3 cell lines investigated herein. Expression levels normalized to β-actin bands for each line are displayed as mean ± SD (n=3). (E) Growth, as normalized to DMSO for the 3 IDH1^*mut*^ glioma cell lines upon 1 μM CB839 for 72 hrs plus 0.1 mM additional Asn. (n=3, bar plots depicting mean ± SD).

**Additional file 5: Figure 5.** (A) Tumor volume of NCH1681-mouse xenograft for 1 week of treatment with CB839 or vehicle. Arrow pointing the starting date of treatment. (B) Glutamate (Glu) and glutamine (Gln) levels obtained from the NMR analysis of tumor tissue. Bar plots depicting the mean ± SD. (C) Representative immunohistochemical images of tumor tissue displaying the upregulation of ASNS in the treated groups for both xenograft models. (D) Quantification of staining of ASNS for tumor tissue (n=3, mean ± SD. *, *p*<0.05 from a t-test). Signal intensity of the slides was computed using ImageJ.

**Additional file 6: Figure 6.** (A) Weight of the tumors extracted from NCH1681-EV/ASNS^*KO*^. (Differences of tumor weights between groups did not attain statistical significance). (B) Asparagine levels obtained from the NMR analysis of tumor tissue. Bar plots depicting the mean ± SD. *, *p*<0.05 from a t-test).

## Data Availability

Any data and materials utilized in this investigation can be available upon request to mioara.larion@nih.gov.

## Abbreviations

ASNS: Asparagine synthetase
EV: Empty vector
GLS: Glutaminase
GPT2: Glutamate-pyruvate transaminase 2
IDH1: Isocitrate dehydrogenase 1
LC-MS: Liquid chromatography mass spectrometry
NMR: Nuclear magnetic resonance
TCA: Tricarboxylic acids
