# Supplementary figures and images for: Metabolic plasticity of IDH1-mutant glioma cell lines is responsible for low sensitivity to glutaminase inhibition
Source: Cancer Metab. 2020 Oct 21;8:23. doi: 10.1186/s40170-020-00229-2 (PMC7579920; doi:10.1186/s40170-020-00229-2)

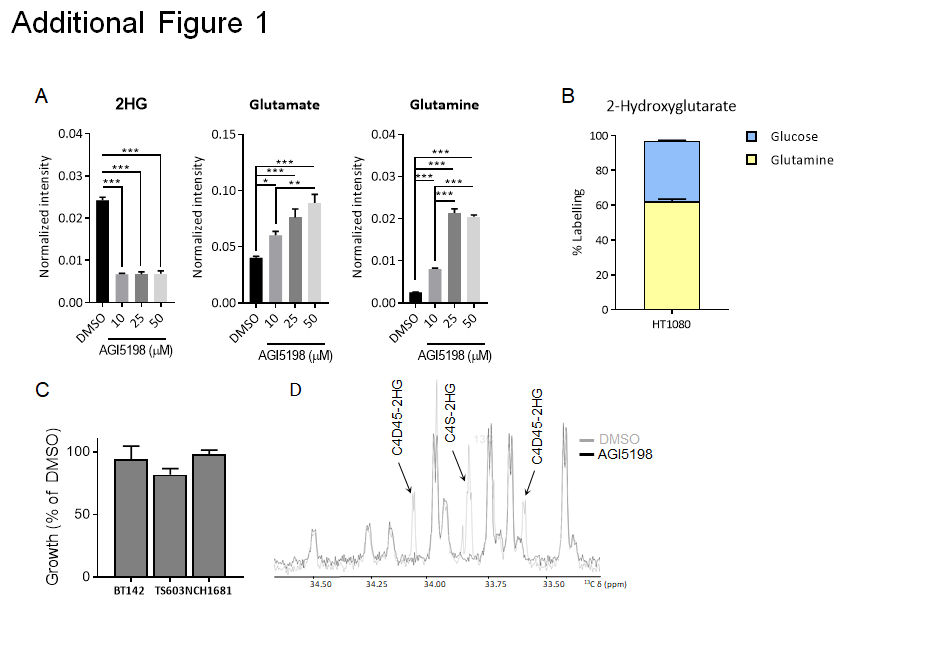

Supplement: Supplementary file 1 — Additional file 1: Figure 1. (A) 2HG, Glutamate and Glutamine levels of HT1080 obtained from the integration of the NMR resonance signals at the 1.80-1.88, 2.33-2.38 and 2.44-2.48 spectral buckets respectively. Intensities were sum-normalized for each spectra. AGI5198 treatment was conducted for 72 hrs. n=3 samples per experiment. *, p<0.05; **, p<0.005; ***, p<0.001 from a one-way ANOVA followed by Tukey's HSD test for multiple comparisons. (B) Different contribution of glucose and glutamine to 2HG in HT1080. 13C tracing experiment performed by LCMS including 13C-U-Glucose or 13C-U-Glutamine for 72 hrs. (C) Viability of IDH1mut glioma cell lines upon 10 μM AGI5198 for 72 hrs. (D) 13C spectral region for 2HG identification including assignments. C4D45, doublet resonance signals at 33.6 ppm and 34.1 ppm from coupling between C4 and C5; C4S, singlet at 33.8 ppm, arising from C4. [file 40170_2020_229_MOESM1_ESM.png]

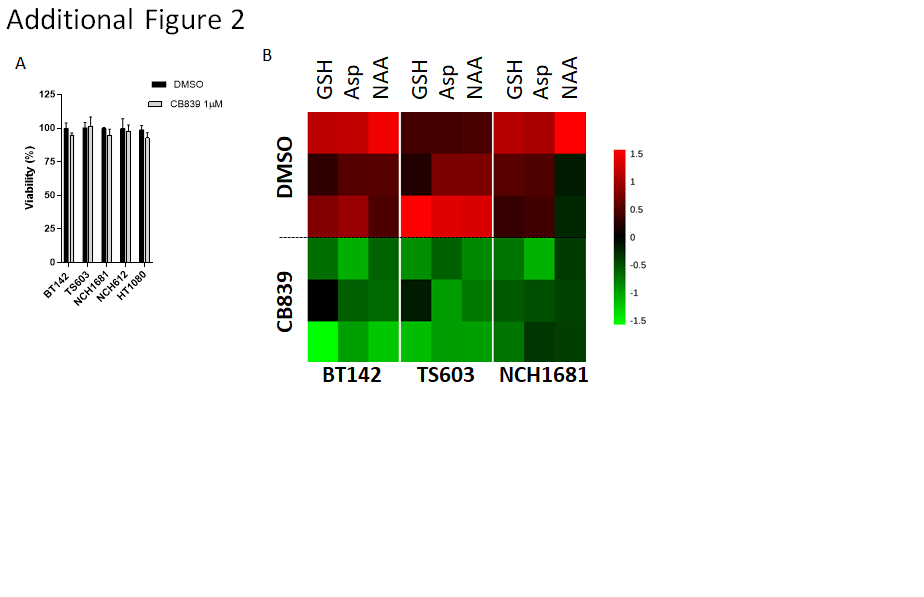

Supplement: Supplementary file 2 — Additional file 2: Figure 2. (A) Viability of IDH1mut glioma cell lines upon 1 μM CB839 for 72 hrs. (B) Levels of glutamate downstream metabolites after treatment with 1 μM CB839 for 72 hrs. [file 40170_2020_229_MOESM2_ESM.png]

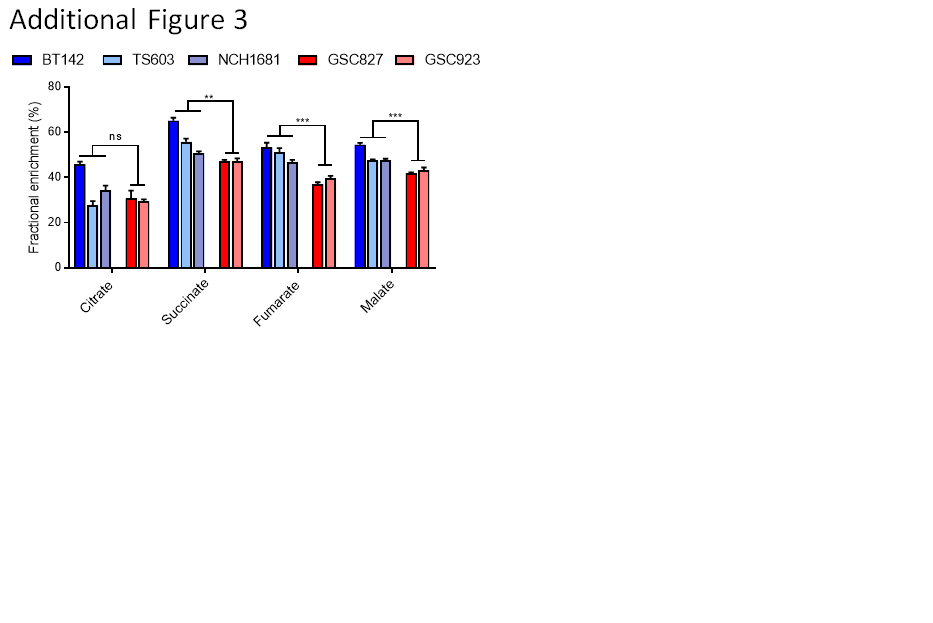

Supplement: Supplementary file 3 — Additional file 3: Figure 3. Fractional enrichment of TCA cycle metabolites in glioma cell lines both IDH mutant and wild type via LCMS. Cells were grown in media containing 13C-U-Glutamine for 72 hrs. n=3, bar plots depicting mean ± SD. Significance markers (ns, not significant; **, p<0.005; ***, p<0.001) arising from a t-test of the averaged percentages of IDH1mut vs IDH1wt. [file 40170_2020_229_MOESM3_ESM.png]

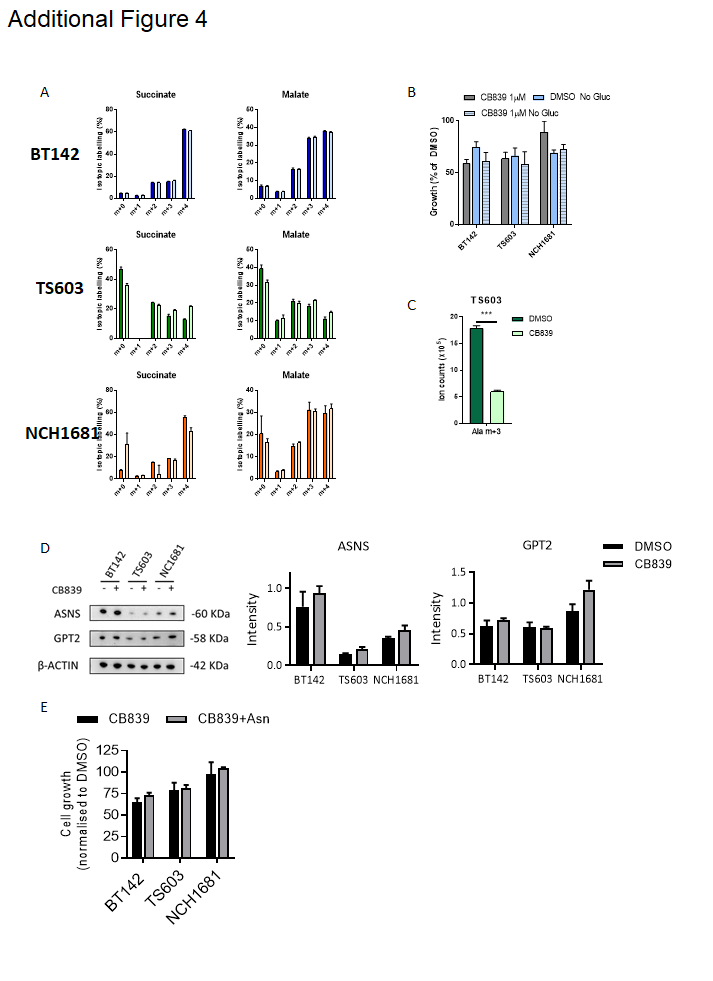

Supplement: Supplementary file 4 — Additional file 4: Figure 4. (A) 13C labelling of TCA cycle metabolites from 13C-U-glucose as isotopologue % over the total pool of the metabolite (n=3, bar plots depicting mean ± SD). (B) Growth of IDH1mut glioma cell lines upon treatment with CB839 and in glucose (gluc) deprivation conditions (n=3, bar plots depicting mean ± SD). (C) Levels of alanine m+3 from 13C-U-glucose in TS603 cell line upon treatment with 1 μM CB839 for 72 hrs (n=3, bar plots depicting mean ± SD, ***, p<0.001, from a t-test). (D) Western Blot of ASNS and GPT2 for the 3 cell lines investigated herein. Expression levels normalized to β-actin bands for each line are displayed as mean ± SD (n=3). (E) Growth, as normalized to DMSO for the 3 IDH1mut glioma cell lines upon 1 μM CB839 for 72 hrs plus 0.1 mM additional Asn. (n=3, bar plots depicting mean ± SD). [file 40170_2020_229_MOESM4_ESM.png]

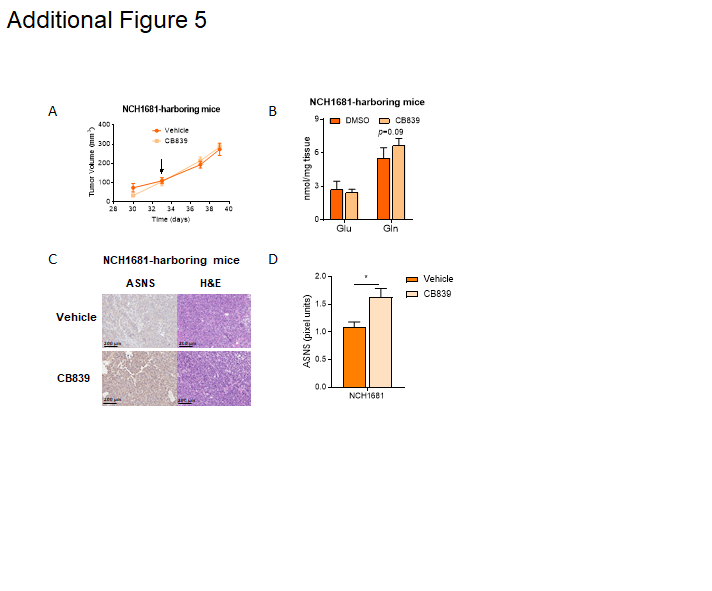

Supplement: Supplementary file 5 — Additional file 5: Figure 5. (A) Tumor volume of NCH1681-mouse xenograft for 1 week of treatment with CB839 or vehicle. Arrow pointing the starting date of treatment. (B) Glutamate (Glu) and glutamine (Gln) levels obtained from the NMR analysis of tumor tissue. Bar plots depicting the mean ± SD. (C) Representative immunohistochemical images of tumor tissue displaying the upregulation of ASNS in the treated groups for both xenograft models. (D) Quantification of staining of ASNS for tumor tissue (n=3, mean ± SD. *, p<0.05 from a t-test). Signal intensity of the slides was computed using ImageJ. [file 40170_2020_229_MOESM5_ESM.png]

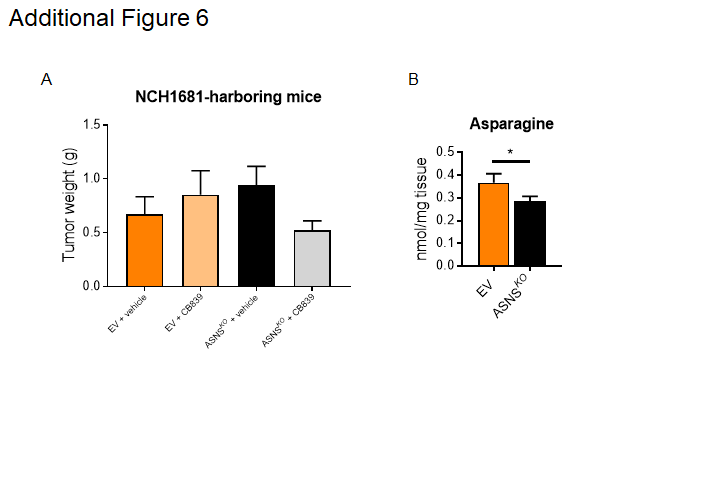

Supplement: Supplementary file 6 — Additional file 6: Figure 6. (A) Weight of the tumors extracted from NCH1681-EV/ASNSKO. (Differences of tumor weights between groups did not attain statistical significance). (B) Asparagine levels obtained from the NMR analysis of tumor tissue. Bar plots depicting the mean ± SD. *, p<0.05 from a t-test). [file 40170_2020_229_MOESM6_ESM.png]
